# Low Mutational Burden of Eight Genes Involved in the MAPK/ERK, PI3K/AKT, and GNAQ/11 Pathways in Female Genital Tract Primary Melanomas

**DOI:** 10.1155/2015/303791

**Published:** 2015-01-28

**Authors:** Kalliopi I. Pappa, George D. Vlachos, Maria Roubelakis, Dimitrios-Efthymios G. Vlachos, Theodora G. Kalafati, Dimitrios Loutradis, Nicholas P. Anagnou

**Affiliations:** ^1^First Department of Obstetrics and Gynecology, University of Athens School of Medicine, Alexandra Hospital, 11528 Athens, Greece; ^2^Laboratory of Biology, Department of Basic Medical Sciences, University of Athens School of Medicine, 11527 Athens, Greece; ^3^Laboratory of Cell and Gene Therapy, Biomedical Research Foundation of the Academy of Athens, 11527 Athens, Greece; ^4^Laboratory of Histopathology and Cytopathology, Department of Pathology, Henry Dunant Hospital, 11527 Athens, Greece

## Abstract

Mucosal melanomas exhibit discrete genetic features compared to cutaneous melanoma. Limited studies on gynecological melanomas revealed significant heterogeneity and low mutational burden. To gain further insight into their genetics and DNA repair efficiency, we systematically investigated the status of eight genes whose products are critically involved in the MAPK/ERK, PI3K/AKT, and GNAQ/11 pathways, including BRAF, NRAS, HRAS, KRAS, c-KIT, PI3K, GNAQ, and GNA11, in a series of 16 primary gynecological melanomas, covering all anatomical locations, ranging from stages I to III. Analysis either by real-time PCR coupled with fluorescence melting curve analysis or by PCR followed by direct sequencing, along with studies for DNA mismatch repair status using immunohistochemistry, disclosed that 15 out of the 16 cases displayed wild-type genotypes, with a single case of vulvar primary melanoma, harboring the activating mutation BRAF^V600E^. Investigations on whether this could reflect partly an efficient mismatch repair (MMR) mechanism were confirmed by normal expression of hMLH1 and hMSH2, suggesting that the lack of mutations could be explained by the operation of alternative pathogenetic mechanisms modulating downstream effectors of the signaling pathways. Our data suggest the presence of additional genetic components and provide the impetus for systematic approaches to reveal these yet unidentified genetic parameters.

## 1. Introduction

Melanocytic malignancies of the female genital tract constitute rare diseases representing only 2-3% of all human malignant melanomas and 18% of all primary mucosal melanomas [1, 2]. Among the various sites of the genital tract, vulvar melanoma exhibits the highest frequency (76.7%), followed by vaginal (19.8%) and cervical melanomas, while uterine and ovarian melanomas are extremely rare [1–7].

Although UV radiation is considered as the main cause of malignant melanoma concerning the sun-exposed body sites [8, 9], it seems that other mechanisms are also capable of initiating melanocyte malignant transformation, leading to tumours with different clinical behaviour. Specifically, malignant melanoma of the female genital tract is biologically aggressive, difficult to manage, carrying a poor prognosis and a high incidence of recurrence, while its pathogenesis is still obscure and to a large extend, independent of UV radiation [10].

There is considerable documentation that the majority of melanomas harbour a variable number of specific genetic changes in key protein kinase signalling pathways, like many other types of cancers. Functional aberrations and mutations in the MAPK/ERK and PI3K/AKT pathways are thought to represent early events in melanocyte transformation with BRAF mutations occurring as early as in benign premalignant nevi [11]. Particularly, BRAF mutations have been identified in about 65% in cutaneous melanomas [12], representing the most common somatic mutation in melanomas, while on the contrary, there is a striking paucity (3–5%) of BRAF mutations in mucosal melanomas in general [12, 13]. Approximately 80% of the detected mutations involve a single substitution in exon 15 of the BRAF gene at position 600, which most commonly substitutes valine for glutamic acid (V600E), designated as BRAF^V600E^ and resulting in permanent activation of BRAF [14].

The protooncogenes encoding the H-Ras, K-Ras, and N-Ras proteins, are also frequently mutated in many human cancers, resulting in a constitutively active state [12]. Although NRAS mutations have been reported in 14% of human melanoma cell lines and 15–25% of melanoma clinical specimens [15–17], nevertheless, HRAS and KRAS mutations are not common in melanoma [12].

Furthermore, PI3KCA activating mutations common in many cancers have been detected in ≤3% of melanomas [18, 19], while recently GNAQ/GNA11 mutations have been detected in uveal melanomas in up to 34–48% [20] compared to <1% of cutaneous melanomas [13]. Finally, c-KIT missense mutations have been reported in 21% of the mucosal, 11% of the acral, and 17% of chronic sun-damaged cutaneous melanomas, while being absent however in non-sun-damaged cutaneous melanomas [21–24].

In contrast to the numerous mutational data in cutaneous melanomas, very limited data are available [23–28], concerning either the full spectrum of mutational events affecting the MAPK/ERK, PI3K/AKT, and GNAQ/11 pathways in female genital tract melanomas or the DNA mismatch repair (MMR) status [29] in the same type of malignancy. To gain insight into the molecular genetics of melanoma of the female genital tract and to its DNA MMR status, in the present study we systematically investigated the mutational status of eight genes whose products are critically involved in the MAPK/ERK, PI3K/AKT, and GNAQ/11 pathways, such as BRAF, NRAS, HRAS, KRAS, c-KIT, PI3K, GNAQ, and GNA11, by employing either real-time PCR coupled with fluorescence melting curve analysis for mutation-specific PCR detection, or PCR followed by direct sequencing techniques, along with studies to determine the DNA MMR status using immunohistochemistry.

## 2. Materials and Methods

### 2.1. Tumor Sample Selection and Classification

Specimens of formalin-fixed and paraffin embedded primary melanomas from patients and control subjects were retrieved from the Departments of Pathology of the Sotiria General Hospital for Chest Diseases, the Henry Dunant Hospital, the Mitera Maternal Hospital, and the Alexandra Hospital, in Athens. A total of 16 melanoma samples along with 3 control samples derived from normal skin, cutaneous melanoma, and metastatic melanoma were evaluated. Original histopathology data for each case were initially obtained by analyzing hematoxylin and eosin (HE)-stained sections from all tumors and the samples were further reviewed by a surgical pathologist for the confirmation of diagnosis. All women were white Caucasians with a mean age of 62.3 ± 17.1 years. The tumors consisted of primary melanomas of the female genital tract with the following anatomic locations: 7 cases of vaginal melanoma (43.7%), 3 cases of vulvar melanoma (18.7%), 4 cases of clitoral melanoma (25.0%), and 2 cases of cervical melanoma (12.5%). Tumor staging was performed according to the International Federation of Gynecology and Obstetrics Classification System for Cervical Melanoma and the revised American Joint Committee on Cancer 2010 tumor-node-metastasis (TNM) melanoma staging for the vulva and vagina [30, 31], while microstaging was estimated according to the Breslow depth, histology type, and growth phase [2].

### 2.2. DNA Extraction

Tissues were sectioned and sections were further microdissected in order to obtain >80% neoplastic cells. Genomic DNA was extracted using the QIAamp extraction DNA kit (Qiagen GmbH, Hilden, Germany) according to the manufacturer's protocols. Concentration of genomic DNA was assessed by a GenQuant spectrophotometer (Pharmacia LKB Biotechnology Inc., Piscataway, New Jersey), as previously described [32].

### 2.3. Mutation Analysis

For the BRAF gene V600E mutational hotspot, a pair of specific primers and two internal oligonucleotide probes were designed [33] and obtained from TIB MOLBIOL Syntheselabor GmbH (Berlin, Germany). The primers for the real-time polymerase chain reaction (PCR) of the BRAF gene (GenBank Accession number AC006344) were BRF-F1 (5′-CCTAAACTCTTCATAATGCTTGCTC-3′) located in intron 15 and BRF-R1 (5′-GACTTTCTAGTAACTCAGCAGCATC-3′) located in intron 14, generating a 263 bp product, as described by Ikenoue et al. [33]. The PCR mixture included also a set of detection and anchor probe [33]. The detection probe DP-BRF 5′-TCGAGATTTCACTGTAGCATC-3′ was 3′-phosphorylated and 5′-labeled with LC-Red 640, while the anchor probe AP-BRF 5′-CAGACAACTGTTCAAACTGATGGGACCCACTCC-3′ located one nucleotide downstream from the detection probe was 3′-labeled with fluorescein isothiocyanate.

Real-time PCR was performed employing the Roche LightCycler 2.0 detection system (Roche Diagnostics GmbH, Mannheim, Germany) in 20 *μ*L volumes in glass capillaries, containing 50 ng of sample DNA in 2 *μ*L, 13.2 *μ*L of H_2_O, 1.6 *μ*L of MgCl_2_ (25 mM/L), 0.4 *μ*L each of BRF-F1 and BRF-R1 primers (25 mM/L each), 0.2 *μ*L each of DP-BRF (40 mM/L) and AP-BRF (20 mM/L) probes, and 2 *μ*L of 10x LightCycler DNA Master hybridization probes (Roche Diagnostics) containing Taq DNA polymerase reaction buffer, a deoxynucleoside triphosphate mixture, and 10 mM/L MgCl_2_ [33]. The cycling conditions consisted of an initial denaturation at 94°C for 2 min, followed by 45 cycles with denaturation at 94°C for 0 s, annealing at 55°C for 10 s, and extension at 72°C for 15 s, with a ramping rate of 20°C/s. Following the amplification process, the fluorescence melting curve analysis was performed. The samples were denatured at 94°C for 0 s, held at 50°C for 5 s, then slowly heated at 80°C at a ramping rate of 0.1°C/s [33], and finally cooled down at 40°C for 60 s. During this process, the decline in fluorescence was monitored continuously and melting curves were constructed automatically by the LightCycler software. The melting curves were converted to melting peaks by plotting the negative derivative of the fluorescence with respect to temperature (−d*F*/d*T*) [33].

Individual mutations of the NRAS [34], HRAS [34], and KRAS [32] genes were investigated using six gene-specific oligonucleotide primer pairs, designed to specifically amplify the regions harboring codons 12-13 in exon 2 and codon 61 in exon 3, followed by direct sequencing, as previously described [32]. The sequences of the six PCR primer pairs utilized were as follows: (a) NRAS 12-13 forward 5′-GCTGGTGTGAAATGACTGAG-3′ and NRAS 12-13 reverse 5′-GATGATCCGACAAGTGAGAG-3′; (b) NRAS 61 forward 5′-CCTGTTTGTTGGACATACTG-3′ and NRAS 61 reverse 5′-CCTGTAGAGGTTAATATCCG-3′; (c) HRAS 12-13 forward 5′-AGGAGACCCTGTAGGAGGA-3′ and HRAS 12-13 reverse 5′-CGCTAGGCTCACCTCTATAGTG-3′; (d) HRAS 61 forward 5′-GTCCTCCTGCAGGATTCCTA-3′ and HRAS 61 reverse 5′-TGGCAAACACACACAGGAA-3′; (e) KRAS 12-13 forward 5′-GTGTGACATGTTCTAATATAGTCA-3′ and KRAS 12-13 reverse 5′-GAATGGTCCTGCACCAGTAA-3′; (f) KRAS 61 forward 5′-TCAAGTCCTTTGCCCATTTT-3′ and KRAS 61 reverse 5′-TGCATGGCATTAGCAAAGAC-3′. The PCR conditions for NRAS, HRAS, and KRAS amplification involved initial denaturation at 95°C for 15 min, 38 cycles of denaturation at 94°C for 30 s, and annealing at 56.5°C for 30 s, followed by an extension step of 72°C for 2 min. The PCR conditions for KRAS 61 were similar except the annealing step which was performed at 50°C for 30 s.

The primer pairs for the amplification of exons 11, 13, 17, and 18 of the c-KIT gene and the PCR conditions employed were as described in detail before [24].

Mutational analysis of the PI3K gene for exons 9 and 20 was performed utilizing the following pairs of oligonucleotide primers and the PCR conditions as described [35]: (a) PI3K exon 9 forward 5′-GATTGGTTCTTTCCTGTCTCTG-3′ and PI3K exon 9 reverse 5′-CCACAAATATCAATTTACAACCATTG-3′; (b) PI3K exon 20 forward 5′-TGGGGTAAAGGGAATCAAAAG-3′ and PI3K exon 20 reverse 5′-CCTATGCAATCGGTCTTTGC-3′.

Analysis for the oncogenic mutations [20, 36] in GNAQ and GNA11 genes in exon 4 (R183) and exon 5 (Q209) was performed using the following primer pairs: (a) GNAQ R183 forward 5′-TGGTGTGATGGTGTCACTGACATTCTCAT-3′ and GNAQ R183 reverse 5′-AGCTGGGAAATAGGTTTCATGGACTCAGT-3′; (b) GNAQ Q209 forward 5′-CCCACACCCTACTTTCTATCATTTAC-3′ and GNAQ Q209 reverse 5′-TTTTCCCTAAGTTTGTAAGTAGTGC-3′; (c) GNA11 R183 forward 5′-GTGCTGTGTCCCTGTCCTG-3′ and GNA11 R183 reverse 5′-GGCAAATGAGCCTCTCAGTG-3′; (d) GNA11 Q209 forward 5′-CGCTGTGTCCTTTCAGGATG-3′ and GNA11 Q209 reverse 5′-CCACCTCGTTGTCCGACT-3′. PCR conditions for their amplification were performed as previously described [20, 36].

All PCR products were analysed by direct DNA sequencing in both directions with GenomeLab DTCS Quick Start Kit, using the Beckman Coulter CEQ 8000 Genetic Analysis System (Beckman Coulter, Inc., Fullerton, CA, USA).

### 2.4. Immunohistochemical Studies

Immunostaining of the melanoma specimens for the expression of the two proteins hMLH1 and hMSH2 involved in the nucleotide mismatch repair (MMR) following DNA replication or repair was performed at room temperature for 30 min, using a mouse monoclonal anti-human MLH1 antibody (Clone ES05 Ready-to-Use) from Dako Denmark A/S (Glostrup, Denmark) and a specific mouse monoclonal antibody against hMSH2 (Clone FE11 Ready-to-Use) also from Dako, following microwave pretreatment of the tissue sections immersed in Envision FLEX Target Retrieval Solution High pH (50x) from Dako. This was followed by a 30 min incubation with Dako REAL Link, Secondary Antibody (LINK), and a 30 min incubation with AP Enzyme (ENHANCER) from Dako. Sections were then incubated with Substrate Working Solution from Dako, consisting of Permanent Red Substrate Buffer and of Permanent Red Chromogen in a 100 : 1 ratio, for 20 min. Finally, the slides were counterstained with hematoxylin, dehydrated in graded alcohol, dried, and cover-slipped.

Specific staining of each antibody was identified primarily in the nucleus; thus, immunoreactivity was evaluated based on the nuclear-positive cells. The data were evaluated independently by two expert dermatopathologists and semiquantitatively estimated [29], based on the percentage of positive cells as follows: negative, absence of nuclear-positive cells; weakly positive (+), <10% positive cells; moderately positive (++), 10–50% positive cells; strongly positive (+++), >50% positive cells. Normal adjacent cells to the melanoma primary site and biopsy specimens from colorectal cancer cases served as internal control.

## 3. Results

### 3.1. Clinicopathological Features

The clinical and pathological features of the 16 cases of female genital tract melanomas are shown in Table 1. None of the patients had a family history for melanoma, while only 5 patients (31.2%) developed nodal or distant metastasis. Regarding clinical staging, five patients (31.2%) were at stage I (A or B), nine patients (56.2%) were at stage II (A–C), and two patients (12.5%) were at stage III (B). The melanomas in the majority of the patients (56.2%) exhibited the thickest Breslow depth of >3 mm (stage V), with melanin pigmentation documented in all cases. The most common histological subtype was represented by nodular melanoma (37.5%), followed by superficial spreading melanoma (31.2%) and mucosal lentiginous melanoma (18.7%) and by a single case of mucosal desmoplastic melanoma, that is, patient 4. Finally, this series of melanoma patients displayed predominantly (62.5%) a vertical pattern of growth phase.

### 3.2. Mutational Analysis of Eight Genes Involved in Melanoma

**Figure 1:**
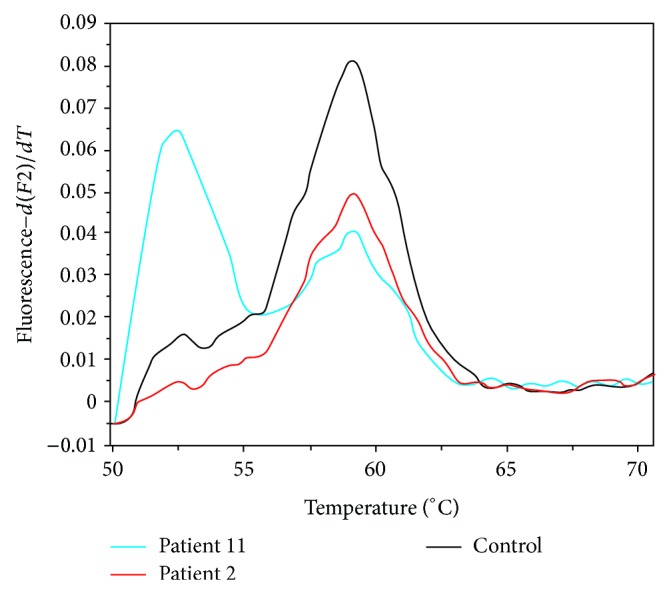
Fluorescent melting curve analysis for the detection of the BRAF^V600E^ point mutation based on the resulting distinct melting temperatures (*T*_*m*_) of duplexes formed between the wild-type probe and the DNA from either the wild-type or the V600E mutant. The melting peaks for the wild-type and the V600E mutant occur at different melting temperatures, that is, 59.2°C and 52.5°C, respectively. The BRAF^V600E^ mutation is shown for patient 11 (blue curve), whose tumor cells exhibit both types of BRAF alleles, since the PCR product shows also a wild-type peak. Patient 2 (red curve) lacks the mutation, exhibiting a single peak, corresponding to the normal control peak (black curve).

Molecular analysis of the hotspots for mutations in eight genes involved in major signaling pathways critically affected in melanoma, employing PCR analysis followed by direct sequencing, disclosed that 15 out of the total 16 cases of female genital tract primary mucosal melanomas displayed wild-type genotypes for exons 2 (codons 12 and 13) and 3 (codon 61) of NRAS, HRAS, and KRAS, for exons 11, 13, 17, and 18 of c-KIT, for exons 9 and 20 of PI3K, and for exons 4 (R183) and 5 (Q209) of GNAQ and GNA11 genes, as shown in Table 2. On the contrary, by employing mutation-specific real-time PCR detection studies coupled with fluorescence melting curve analysis, we identified a single case with vulvar superficial spreading primary melanoma, designated as patient 11 in Table 1, harboring the activating mutation BRAF^V600E^ in exon 15 of BRAF, as shown by the additional abnormal peak with a melting temperature of 52.5°C, besides the normal peak with a melting temperature of 59.2°C (Figure 1).

### 3.3. Studies for the Status of MMR Mechanism

In view of the documented paucity of mutations of the eight genes in our series, we investigated whether this finding could reflect the operation of an efficient mismatch repair (MMR) mechanism in this type of mucosal melanomas. To this end, we evaluated by immunohistochemistry the expression of two major MMR proteins, hMLH1 and hMSH2. Immunohistochemical staining for hMLH1 and hMSH2 was evaluated in 11 representative cases.

**Figure 2:**
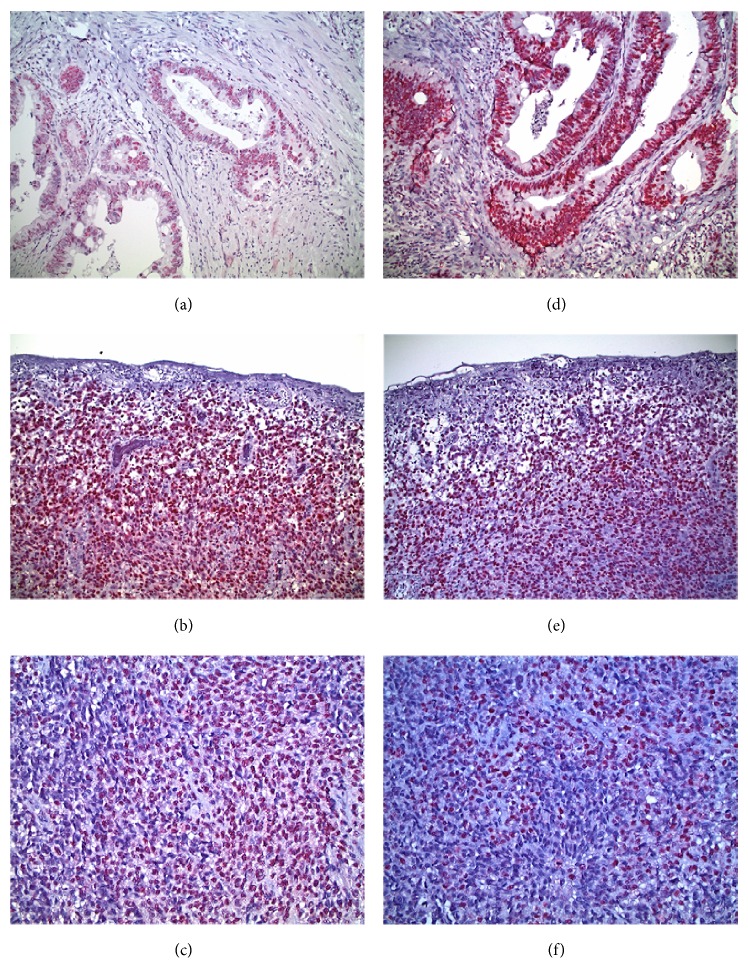
Immunohistochemistry analysis for the expression of the hMLH1 and hMSH2 MMR proteins in mucosal melanomas. (a) MMR-proficient/BRAF mutant colorectal cancer control exhibiting positive nuclear staining for hMLH1. (b) Vertical growth phase mucosal melanoma with ulcerated surface. Almost all melanoma cells display intense nuclear staining for hMLH1. (c) Mucosal melanoma with spindle and epithelioid cells strongly positive for hMLH1. (d) MMR-proficient/BRAF mutant colorectal cancer control displaying positive nuclear staining for hMSH2. (e) Vertical growth phase mucosal melanoma with ulcerated surface and diffuse nuclear staining for hMSH2. (f) Mucosal melanoma with spindle and epithelioid cells strongly positive for hMSH2. Photomicrographs (a), (b), (d), and (e) are of ×20 magnification, and photomicrographs (c) and (f) are of ×40 magnification.

For the hMLH1 protein, staining was weakly positive (+) in 1 case (9.1%); moderately positive (++) in 4 cases (36.4%); and strongly positive (+++) in 6 cases (54.5%), as shown in Figures 2(b) and 2(c). For hMSH2, staining was weakly positive (+) in 2 cases (18.2%); moderately positive (++) in 3 cases (27.3%); and strongly positive (+++) in 6 cases (54.5%). Representative results are shown in Figures 2(e) and 2(f). Thus, moderate to strong positive hMLH1 staining was assessed in 90.9% of the cases, while a similar pattern of positive staining for hMSH2 protein was documented in 81.8% of the cases. A comparative semiquantitative pattern of positive staining was also observed both in normal cells adjacent to the melanoma cells, but also in specimens of MMR-proficient/BRAF mutant colorectal cancer cells, serving as controls (Figures 2(a) and 2(d)). Therefore, these data suggest that the lack of activating mutations of the eight genes is compatible with a detectable efficient MMR status involving hMLH1 and hMSH2 proteins and imply the presence of alternative pathogenetic mechanisms operating in this type of mucosal melanomas.

## 4. Discussion

In the present study, we undertook a comprehensive molecular approach to delineate the genetic heterogeneity of mucosal melanomas and particularly those of primary melanomas of the female genital tract. Due to the limited and fragmentary studies focusing especially on oncogenic mutations in gynecological melanomas, we opted to systematically investigate—in a series of 16 well-characterized primary melanomas of the female genital tract—the mutational status of eight genes whose products are the primary effectors critically involved in the MAPK/ERK, PI3K/AKT, and GNAQ/11 pathways, such BRAF, NRAS, HRAS, KRAS, c-KIT, PI3K, GNAQ, and GNA11, employing either real-time PCR coupled with melting curve analysis for mutation-specific detection of BRAF or PCR followed by direct sequencing, along with studies on the MMR status using immunohistochemistry. Our data provide several new features of potential importance on the pathogenesis of the gynecological melanomas.

Recent combined data analysis from various reports so far has disclosed a prevalence of 5% for BRAF and 14% for NRAS oncogenic mutations in all types of mucosal melanomas [37], strikingly in contrast to the BRAF prevalence of 56–59% in cutaneous melanomas. These frequencies are even lower in the subset of mucosal melanomas of the female genital tract. Specifically, in a study involving 30 patients with vulvar and vaginal melanomas, BRAF and NRAS mutations exhibited a 7% and 10% frequency, respectively [24], while similar low frequencies of BRAF mutation ranging from 0% to 12.5% have been detected in other studies [26, 27, 37]. Furthermore, the recently calculated [38] cumulative published frequencies of the oncogenic mutations in primary melanomas of the female genital tract are consistent with the notion that gynecologic melanomas exhibit frequently c-KIT mutations (26%) and harbor less frequently NRAS mutations (15%), while BRAF mutations remain very uncommon (5%). The latter finding is consistent with our data, where BRAF mutation exhibited a 6.2% frequency. It is conceivable that the deviations of the reported frequencies of the oncogenic mutations among the limited studies so far might reflect primarily differences in the mutation screening techniques and strategies employed.

Regarding the other oncogenic mutations that have been described in either cutaneous or mucosal melanomas, c-KIT missense mutations and/or copy number amplifications occur in about 21% of mucosal melanomas, while they are absent in non-sun-damaged cutaneous melanomas [21–24, 38]. However, sequence analysis of the common hotspot mutation sites of c-KIT exons 11, 13, 17, and 18 in our series revealed no mutations. Similar low-burden mutation status of c-KIT has been reported previously [23, 24]. On the other hand a recent three-center study of a series of 65 cases of vulvar and vaginal melanomas, detected no mutations of BRAF, a 12% mutation frequency of NRAS, and c-KIT amplification at the same frequency, while c-KIT mutations appeared to be specific for vulvar melanomas (18%), since they were undetected in vaginal melanomas [39], confirming the findings of a previous study [24]. The latter finding reveals similarities of the vaginal melanomas to esophageal melanomas, which also lack c-KIT mutations but may harbor NRAS alterations [40]. Furthermore, in the latter study, involving a series of 10 esophageal melanomas, similar patterns of relatively low mutation burden of five genes involved in the major signaling pathways—consistent with our data—have been documented regarding c-KIT (20%), KRAS (10%), and BRAF (10%), while there was absence of mutations for NRAS and PDGFR genes [40].

**Figure 3:**
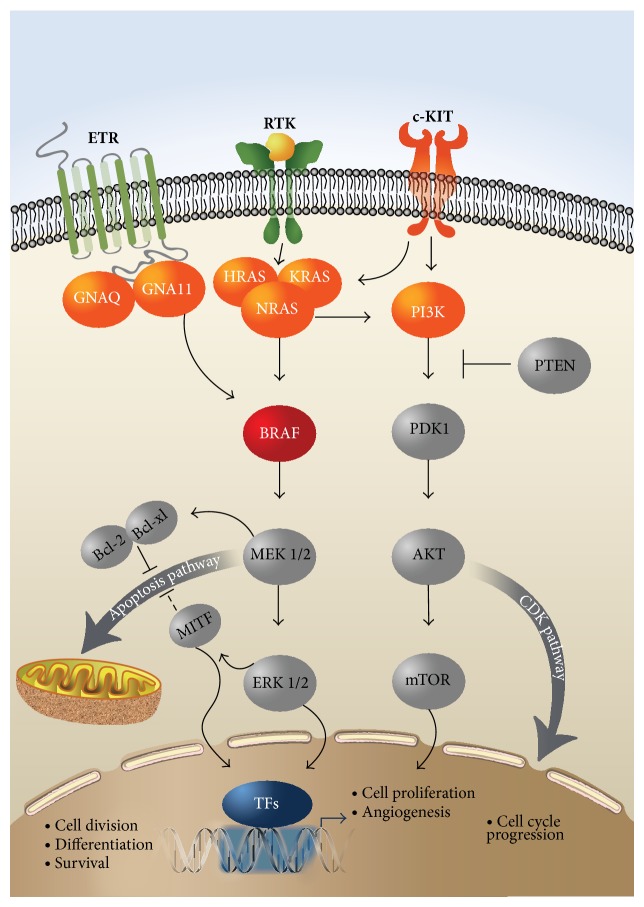
The individual involvement of the products of the eight studied genes (shown in red colour) in the (a) RAS/RAF/MEK/MAPK, (b) PI3K/AKT, and (c) GNAQ/11 signalling transduction pathways. BRAF (shown in dark red colour) was the only gene found mutated in a single patient in our series. All three members of the RAS family of GTPases, that is, the HRAS, KRAS, and NRAS, function as regulated binary switches and, following growth factor stimulation via the receptor tyrosine kinase (RTK), bind and activate downstream effectors, mainly BRAF and PI3K, leading sequentially to the activation of the ERK and AKT kinases, operating in the two major signal transduction pathways, respectively. Additional pathways, such as the apoptosis pathway and the CDK pathway leading to cell cycle progression, can be further activated from these downstream effectors of the two major pathways. Upstream of RAS and PI3K, c-KIT can be also mutated or amplified in melanoma and in turn activate the downstream effectors of the two major pathways. An alternative pathway via the endothelin receptor (ETR), involving two heterotrimeric G-*α*-proteins, that is, GNAQ and GNA11, can also activate the BRAF/MEK/MAPK/ERK pathway and appears to be important for both normal melanocyte development and melanoma formation. Finally, MITF plays also an essential role in melanocyte-specific transcription, exhibiting multiple regulatory functions. mTOR, mammalian target of rapamycin; MITF, microphthalmia-associated transcription factor; TFs, transcription factors.

The recently identified GNAQ^Q209^ and GNA11^R183^ mutations affecting the two effectors of the third signaling pathway (Figure 3) are also considered potential drivers of MAPK pathway activation. However, no mutations of the two genes, analyzed for the first time in gynecological melanomas, were detected in our series. The characteristic feature of these mutations is their predominant occurrence in uveal melanomas in up to 85% of the cases [41, 42], while being nearly absent in cutaneous melanomas or in other subsets of the disease [43]. These putative driver mutations, like the BRAF versus NRAS mutations, exhibit also a mutually exclusive pattern. In contrast to uveal melanomas, no such mutations have been documented in mucosal melanomas [37] and particularly in female genital tract melanomas, as in our series. A single exception has been recently reported [44] concerning a case of metastatic mucosal melanoma harboring the GNAQ^Q209P^ mutation in exon 5, with no mutations of the BRAF, NRAS, or c-KIT genes.

These two most common and mutually exclusive activating mutations of BRAF^V600E^ and NRAS^Q61K^ have been primarily documented in more than 60% of human melanomas, leading to the constitutive signaling of the mitogen-activated protein kinase (MAPK) pathway [12, 15–17], as shown in Figure 3. Although the ensuing altered MAPK signaling has been correlated with tumor growth, other studies on long-term silent human or transgenic zebrafish [45] nevi harboring such mutations have implied that these driver mutations are necessary but not sufficient for the progression of malignant melanoma, suggesting the operation and requirement of additional alternative somatic events involving the contribution of many different mutated genes [45–47]. Also the fact that, in about 50% of the wild-type mucosal melanomas and particularly of vulvar and nonvulvar melanomas [24], both RAF/MEK/ERK and PI3K/AKT pathways are actually activated strongly suggests the presence of alternative mechanisms—other than activating mutations in the c-KIT, NRAS, and BRAF genes—which can lead to constitutive activation of the ERK and AKT pathways and eventually to melanomagenesis. Such alternative mechanisms could include oncogenic activation of the PI3K gene which however in our series was also found to be nonmutated or the recently documented [48] divergent roles of the oncogenic and of the remaining wild-type Ras family members, in the regulation of the MAPK signaling.

The advent of the whole human genome or exome analysis technologies and their application in human melanomas have revealed a wealth of novel mutations associated with its pathogenesis [46, 49, 50]. Thus, employing for the first time whole genome and whole exome sequencing on the genomes of 10 mucosal melanomas—including four gynecological melanomas—it was documented that the somatic mutation rates were considerably lower compared to sun-exposed cutaneous melanoma, but comparable to the rates seen in cancers not associated with exposure to known mutagens [50]. These alterations were associated also with substantially more copy number and structural variations, compared to those of cutaneous melanomas. The cause of this chromosomal instability in mucosal melanomas is not yet clear, although there were no recurrent mutations in genes involved in DNA repair and genome integrity, consistent with our data on the efficient expression of hMLH1 and hMSH2 mismatch repair (MMR) proteins. These novel findings of the omics technologies convincingly document the distinct genetic features of the two major entities, that is, cutaneous versus mucosal melanomas, and suggest that different pathogenetic mechanisms with structural variations are playing a more important role in mucosal melanomas [50].

Furthermore, refined analysis of the initiating driving mutations in the absence of UV mutagenesis in zebrafish models, mimicking the setting of mucosal melanomas, revealed striking genetic heterogeneity, genotype-specific mutation patterns, and a potential novel pathway to BRAF-driven tumorigenesis [51]. These recent findings provide actually novel insights into the molecular events of melanoma pathogenesis in the context of the low mutational burden, as it occurs in our series of primary mucosal melanomas of the female genital tract. Specifically, an inactivating mutation (V170fs)—in the absence of BRAF mutation—was documented in the* pi3kip1* gene, whose product in humans binds to the p110 catalytic subunit of PI3K, downregulating indirectly its activity, thus underscoring the putative role of PI3K cooperation with MAPK deregulation in human melanoma [52] (Figure 3). However, in our studies, all 16 samples have disclosed absence of any mutations in exons 9 and 20 of the PI3K gene, implying the presence of functional PI3K alleles. These UV-free engineered melanomas have also displayed an absence of transcriptomic signatures of repair processes [51], a fact consistent with the absence of a detectable defective mismatch repair (MMR) mechanism involving the hMLH1 and MSH2 proteins in our series. On the contrary, complete or partial loss of these two MMR proteins has been documented to occur in a subset of dysplastic nevi and cutaneous melanoma [53].

In our study, the hypothesis of whether this extensive lack of oncogenic mutations in our series could be attributed in part to an efficient MMR mechanism was investigated by focusing on the status of hMLH1 and hMSH2 proteins of the DNA mismatch repair system, which stabilize the genome and repair postreplicatively the mismatches and the small single stranded DNA loops [54]. Our assessment confirmed an efficient MMR status, suggesting that the lack of activating mutations could be explained by the operation of alternative pathogenetic mechanisms either involving aberrations of copy number and structural variations [50] and/or modulating further downstream effectors of the signaling pathways [24, 51].

## 5. Conclusions 

In summary, our informative data along with the above recent developments [50, 51] suggest the presence of alternative pathogenetic mechanisms operating in mucosal melanomas and particularly in gynecological melanomas and provide the impetus for additional systematic approaches to reveal these yet unidentified genetic parameters, by combining omics technologies employing mRNA, miRNA, and DNA sequencing, integrated with copy number, methylation, and proteomic analysis, following the recent successful generation of the comprehensive molecular profiling of lung adenocarcinoma [55].

## Figures and Tables

**Table 1:** Clinicopathological characteristics of the 16 female genital tract primary melanomas.

Patient number	Age	Race	Primary	Location	Melanoma family history	Nodal metastasis	Stage	Breslow depth	Pigmentation	Histology type	Growth phase
1	42	White	Yes	Cervix	No	No	IB1	V	Positive	Mucosal on nevi	Vertical
2	55	White	Yes	Cervix	No	No	IA2	V	Positive	Superficial spreading	Focal vertical
3	34	White	Yes	Clitoris	No	No	IIB	IV	Positive	Mucosal lentiginous	Vertical
4	75	White	Yes	Clitoris	No	No	IIA	III	Positive	Mucosal desmoplastic	Not available
5	53	White	Yes	Clitoris	No	Lung	IIB	V	Positive	Nodular	Vertical
6	86	White	Yes	Vagina	No	No	IIC	V	Positive	Nodular	Vertical
7	95	White	Yes	Vagina	No	No	IB	I	Positive	Mucosal lentiginous	Vertical
8	60	White	Yes	Vagina	No	No	IIA	V	Positive	Nodular	Vertical
9	68	White	Yes	Vagina	No	No	IIC	V	Positive	Nodular	Vertical
10	52	White	Yes	Vulva	No	Nodes	IA	I	Positive	Superficial spreading	Focal vertical
11	64	White	Yes	Vulva	No	No	IIC	V	Positive	Superficial spreading	Focal vertical
12	48	White	Yes	Vulva	No	Nodes and leg	IIIB	V	Positive	Nodular	Vertical
13	44	White	Yes	Vulva	No	No	IIA	II	Positive	Superficial spreading	Not available
14	66	White	Yes	Vulva	No	Nodes	IIIB	II	Positive	Nodular Pagetoid	Vertical
15	70	White	Yes	Vulva	No	Nodes	IA	I	Positive	Superficial spreading	Not available
16	85	White	Yes	Vulva	No	No	IIC	V	Positive	Mucosal lentiginous	Vertical

**Table 2:** Molecular characterization of the eight genes in the 16 female genital tract primary melanomas.

Gene	Location	Number of mutations	%
*BRAF *	Exon 15, V600E	1	6.2%

*NRAS *	Exon 2, codons 12-13	0	0%
	Exon 3, codon 61	0	0%

*HRAS *	Exon 2, codons 12-13	0	0%
	Exon 3, codon 61	0	0%

*KRAS *	Exon 2, codons 12-13	0	0%
	Exon 3, codon 61	0	0%

*cKIT *	Exon 11	0	0%
	Exon 13	0	0%
	Exon 17	0	0%
	Exon 18	0	0%

*PI3K *	Exon 9	0	0%
	Exon 20	0	0%

*GNAQ *	Exon 4, R183	0	0%
	Exon 5, Q209	0	0%

*GNA11 *	Exon 4, R183	0	0%
	Exon 5, Q209	0	0%
